# Effects of chloride ions on corrosion of ductile iron and carbon steel in soil environments

**DOI:** 10.1038/s41598-017-07245-1

**Published:** 2017-07-31

**Authors:** Yarong Song, Guangming Jiang, Ying Chen, Peng Zhao, Yimei Tian

**Affiliations:** 1 School of Environmental Science and Engineering, Tianjin University, Tianjin, 300350 China; 2 Advanced Water Management Centre, The University of Queensland, St. Lucia, Brisbane QLD 4072 Australia; 3 Tianjin Engineering Center of Urban River Eco-Purification Technology, Tianjin, 300350 China

## Abstract

Chloride is reported to play a significant role in corrosion reactions, products and kinetics of ferrous metals. To enhance the understanding of the effects of soil environments, especially the saline soils with high levels of chloride, on the corrosion of ductile iron and carbon steel, a 3-month corrosion test was carried out by exposing ferrous metals to soils of six chloride concentrations. The surface morphology, rust compositions and corrosion kinetics were comprehensively studied by visual observation, scanning electron microscopy (SEM), X-Ray diffraction (XRD), weight loss, pit depth measurement, linear polarization and electrochemical impedance spectroscopy (EIS) measurements. It showed that chloride ions influenced the characteristics and compositions of rust layers by diverting and participating in corrosion reactions. α-FeOOH, γ-FeOOH and iron oxides were major corrosion products, while β-Fe_8_O_8_(OH)_8_Cl_1.35_ rather than β-FeOOH was formed when high chloride concentrations were provided. Chloride also suppressed the decreasing of corrosion rates, whereas increased the difficulty in the diffusion process by thickening the rust layers and transforming the rust compositions. Carbon steel is more susceptible to chloride attacks than ductile iron. The corrosion kinetics of ductile iron and carbon steel corresponded with the probabilistic and bilinear model respectively.

## Introduction

Corrosion of ferrous metals in soil is one of the major causes of durability problems of water, sewage, oil and gas distribution systems. For example, Norin^1^ in 1998 reported that soil corrosion was the fundamental cause of deterioration of underground pipelines. Kirmeyer *et al*.^2^ in 1992 noted that 48% of water pipes were ferrous metals (19% ductile iron) which were regarded as the most susceptible to soil corrosion. The recent investigation revealed that 70% of water pipelines in Australia are ferrous metals buried in soil environments^3^. It is thus important to understand the corrosive environment in soils for ferrous metals. However, the corrosion mechanisms for different soil types still require further analysis due to complex soil natures. Saline soil, in particular, is extremely corrosive to ferrous metal pipelines mainly due to the abundant chloride contents, one of the most substantial natural pollutants in saline-alkaline soils^4^.

Corrosion of ferrous metals in soil is a multiscale process initially induced by the localized electrochemical reactions outlined by Dension *et al*.^5^ in 1932, and further developed by Rossum^6^ in 1969. The electrochemical process is highly influenced by the development of rust oxide layers as well as the film/droplet formation on the metal surface, which are in turn controlled by local environments, i.e. moisture, oxygen, temperature, soluble salts and so forth^7^. The study of those factors influencing the corrosion of ferrous metals in soils has a long and substantial history. However, few of them led to a detailed understanding of the effects of chloride ions on the corrosion of ferrous metals in soil environments. Chloride ions, minor in radius, may be adsorbed or penetrate easily through the passive film even the oxide layer thus damaging their integrity and accelerating the electrochemical reactions afterwards^8^. Besides, the abundant chloride concentrations, conducive to reducing soil resistivity, also indirectly facilitate the electrochemical reactions.

A great number of investigations have demonstrated that chloride ions remarkably influence the composition and protective efficiency of rust layers on ferrous metal surfaces. High concentrations of chloride were reported to induce the production of akaganite (β-FeOOH)^9–12^, which was able to exhibit high reduction reactivity. Asami *et al*.^13^ verified the major accumulation of β-FeOOH in the thick parts of rust layers that served as a Cl^−^ container; β-FeOOH made the layer more porous and accelerated the corrosion process as well. Further, as chloride concentrations increased, the β-FeOOH increased simultaneously^14^. Ma *et al*.^15^ not only speculated that high chloride deposition led to the formation of β-FeOOH, but also reported that low chloride facilitated the accumulation of lepidocrocite (γ-FeOOH). The following transformation from γ-FeOOH to α-FeOOH was more stabilized thus protecting the metal against further corrosion. Most of these conclusions were drawn in the context of atmospheric or water based (marine) environments. For soils, however, the nature and transformation of corrosion products might be quite different due to the heterogeneous and complex soil characteristics. According to Cole and Marney^7^, a detailed investigation of the composition and interaction within iron oxides that develop on ferrous metals in soil has not been undertaken.

On the other hand, the effects of chloride ions on the corrosion kinetics are also controversial. Allam *et al*.^16^, focusing on atmospheric situations, revealed that chloride ion only functioned during the corrosion initiation and failed to penetrate through the thick rust layer at later stages. This phenomenon is in accord with the results presented by Ma *et al*.^15^ that corrosion rates increased initially and then declined with the exposure time in marine atmosphere. Interestingly, Morales *et al*.^17^ further argued that chloride ions might impair the corrosion rate when reaching a certain degree and be considered non-corrosive afterwards, postulating the existence of critical chloride concentrations. Collectively speaking, previous studies failed to support a clear correlation between the chloride concentration and corrosion rates, especially for the corrosion process in soil environments.

Although considerable investigations have studied the effects of chloride on ferrous metals corrosion in atmospheric or aqueous environments, whether chloride ions play an analogous role in soil environments is still ambiguous in literature. Liu *et.al*.^18^ used solutions to simulate soil conditions and carried out electrochemical tests on carbon steel. Although the aggressiveness of the added cations and anions were evaluated, the effects of chloride ions were still not delineated in details. Nie *et al*.^19^ examined the carbon steel corrosion in salty test soils with the chloride concentration as high as 1.41 wt.%. However, it only emphasized the essential role of dissolved oxygen transfer, but neglected the potential influence of abundant chloride ions. Similar situations prevail in traditional soil studies that mainly focus on other common parameters, i.e. soil resistivity, moisture, pH, oxygen diffusion, redox potential and so forth^7, 20, 21^. It is thus essential to clarify the specific effects of chloride ions on the corrosion of ferrous metals in saline soils usually containing more than 0.6 wt.% of chloride ions.

The main objective of this study is to enhance the understanding of correlations between chloride ions and the corrosion processes of ferrous metals i.e. ductile iron and carbon steel in soil environments. Particularly, we aim to determine how the corrosion processes, products and kinetics are affected by chloride ions during the initial stage of corrosion. According to Singh *et al*.^22^, the long-term corrosion rates are largely controlled by the types of rusts formed during the initial stages of exposure of their virgin surfaces. Thus the initial exposure data are very important to predict the corrosion performance. To accelerate the corrosion process during a reasonable time, experiments were carried out by exposing ductile iron and carbon steel to soils of six different chloride concentrations at the temperature 40 °C for 3 months. The effects of chloride ions on the corrosion of ferrous metals were quantified and analyzed by surface characterization, weight loss, pit depth and electrochemical measurements. The results are of great importance for protecting underground pipelines and predicting the potential pipe serviceability especially in chloride enriched soil environments.

## Materials and Methods

### Materials

The ductile iron (QT400-17) and carbon steel (Q235) commonly used for underground pipelines were considered for this study. The standard coupons as described by the Technical Conditions^23^ were utilized in dimensions of (50.0 ± 0.1) × (25.0 ± 0.1) × (2.0 ± 0.1) mm and chemical compositions were shown in Table 1. Each coupon was polished (using a series of waterproof abrasive papers of 500, 800, 1000, and 1200 grit), degreased in acetone, dehydrated in absolute ethanol, dried, weighed and stored in the desiccators. For the electrochemical study, the same types of metals were further fabricated to the working electrodes with a working area of 1 cm^2^. The area experienced the similar treatments to the coupons except for being weighed.

**Table 1:** Chemical compositions of the ductile iron and carbon steel (wt.%).

Metal Types	C	Si	Mn	S	P
Ductile Iron (QT400-17)	3.7	1.55	0.50	0.025	0.06
Carbon Steel (Q235)	0.116	0.30	0.40	0.045	0.045

The soil samples were extracted from 1 m depth underground (where pipes are usually buried) in a field in Tianjin (China). The soil samples were characterized as received according to the National Standards^24^, and the average values were summarized in Table 2. After natural drying and sieving (10 meshes), the soils were stored in the oven at 105 °C to maintain stable and dry. Before exposure experiments, the soil samples were prepared at a controlled moisture content of 20 wt.% identical to its original status. We achieved this preparation by incorporating quantitative amounts of distilled water (Supplementary Table S1) to the soil samples which were pre-dried and pre-weighted. In order to clarify the effects of chloride ions and accelerate the corrosion process, different amounts of sodium chloride were mixed into the distilled water and then evenly dispersed into the soil samples, thus resulting as six soil samples of separate chloride contents, i.e. 0.015%, 0.065%, 0.115%, 0.315%, 0.515%, 1.015% (wt.%). The soil containing 0.015 wt.% of chloride ions was the original soil without extra chloride additions that acted as the control.

**Table 2:** Main parameters of the soil as received from the field in Tianjin (northeastern China).

Parameters	Value	Parameters	Value
Chloride (wt.%)	0.0148	Redox potential (mV)	561
Sulfate (wt.%)	0.0684	Sulfide (wt.%)	0.0192
Resistivity (Ωcm)	2871	Moisture content (wt.%)	20
pH	8.8		

**Figure 1:**
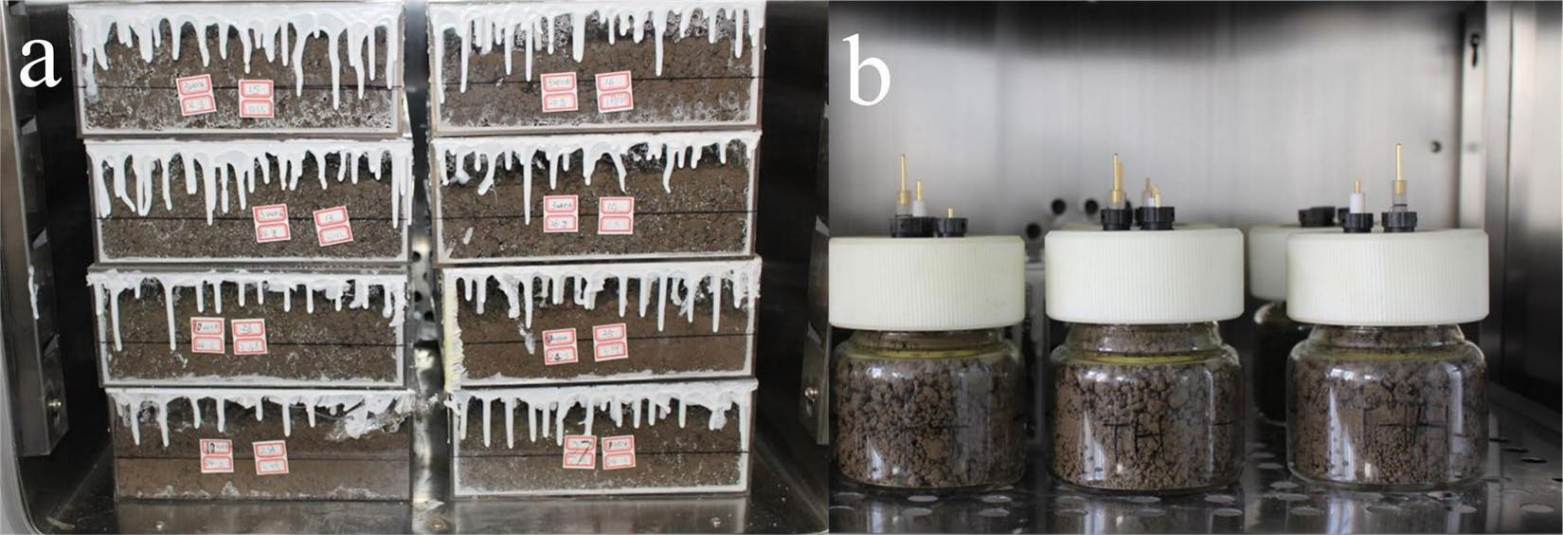
(**a**) The labeled soil reactors filled with soil samples burying coupons; (**b**) the electrochemical cell filled with soil samples.

To simulate the soil environment, the plastic soil reactors (Fig. 1a) and the specially designed electrochemical cells (Fig. 1b) were prepared for the exposure. Prior to be filled with soils, each reactor composed of 5 parallel boxes (5 × 80 × 80 × 200 mm) was cleaned, dried and re-sealed on every seam inside the boxes using silica gel 704. As for the electrochemical system, the cell was roughly 8 cm in diameter, 10 cm in height and 6 cm in diameter of the bottle neck. The counter electrode was a platinum column. The self-designed reference electrode^25^ (Supplementary Fig. S1) used the silver-silver chloride. Also, the traditional electrolyte of saturated KCl in the salt bridge was innovatively replaced by saturated KNO_3_ added with the curing agent; consequently the semisolid state was more stable and exempt from the leakage of electrolyte as well as the interference of extra chloride ions. The self-designed soil reactors and electrochemical cells were demonstrated to be efficient in reducing the water evaporation from soils.

### Exposure tests

The exposure experiments were carried out concurrently both in the soil reactors and the electrochemical cells described above. For six different chloride levels and two types of ferrous metals, i.e. ductile iron and carbon steel, twelve soil reactors were prepared and labeled. In each reactor, five sets of metal coupons, with three coupons in each set, were vertically buried in line in each box of the reactor, in the manner that the space between two adjacent coupons was 50 mm and the coupon bottom was 15 mm away from the bottom of the box. All soil samples in the reactors were filled and compacted in the same method to achieve an identical porosity. After burying the coupons into soils, each reactor was sealed using the plastic cover and the silica gel 704; then they were stored in the constant temperature incubator at 40 °C, which was intended for accelerating the corrosion process according to the Environmental Acceleration Method^26^. Relatively high temperature mainly facilitates the diffusion process instead of changing the corrosion mechanism dramatically^27, 28^. Benmoussa *et al*.^29^ reported that the steel corrosion in soil simulation solution increased with temperature in the range from 20 to 60 °C.

One set of coupons (3coupons) were retrieved from each box for analysis after 1, 3, 5, 7, 12 weeks of exposure. For each sampling event, all coupons were used to characterize the morphology and compositions of the rust layer as well as weight loss measurements. Weight loss measurements were performed by eliminating the corrosion products covering the steel coupons using successive cleanings in hydrochloric acid aqueous solution (3.5 g hexamethylenetetramine + 500 ml distilled water + 500 ml HCl)^15, 30, 31^, until no significant weight change was observed. Also, the coupons retrieved after 12 weeks were conducted with the pit depth measurement by DDC- II Pitting corrosion tester (Xiangwei, China), of which the measurement range is 0–5 mm. For each coupon, 15 pits were randomly selected and measured^32^.

In addition, the exposure of the produced working electrodes to soils in the electrochemical cells was carried out simultaneously. Twelve cells were prepared for two types of working electrodes using ductile iron and carbon steel respectively; each cell was intended for one working electrode exposed to a certain soil condition. Likewise, the electrochemical cells were retrieved from the constant temperature incubator for the electrochemical measurements after 1, 3, 5, 7, 12 weeks of exposure.

### Characterization of rust layers

At the end of each exposure period, the macro-level corrosion morphology of each coupon retrieved from the soil boxes was photographed for visual analysis. Then, the corrosion products covering the coupons were scraped from the coupon surface to characterize the micro morphologies using SEM (Nanosem 430, America). The rust phase was also ground to fine powder samples in a mortar using a pestle for XRD analysis (D/MAX-2500X), which was 18 kw intensity, 2.0◦/min^−1^ scanning speed, and 2 Θ = 10–90° of range using a Cu target.

### Electrochemical measurements

All electrochemical measurements were performed in the cell described above (Fig. 1b) by being connected to an electrochemical workstation (CS350, KeSiTe, China). Prior to each measurement, it generally took 50 min to obtain a steady open circuit potential (*E*_oc_). The scan of the linear polarization measurements were carried out over a range of −15 to 15 mV versus the *E*_oc_ at the scan rate of 0.5 mV/s. The electrochemical impedance spectroscopy (EIS) was performed in the frequency range between 0.01 Hz and 100 kHz with a 10 mV amplitude signal at open circuit. Zview2.0 was used to collect the EIS data, and Cview2.0 was utilized to analyze the polarization curve data. All the electrochemical measurements were conducted at around 25 °C (after the cells cooled down).

## Results and Discussion

### Morphology observation

**Figure 2:**
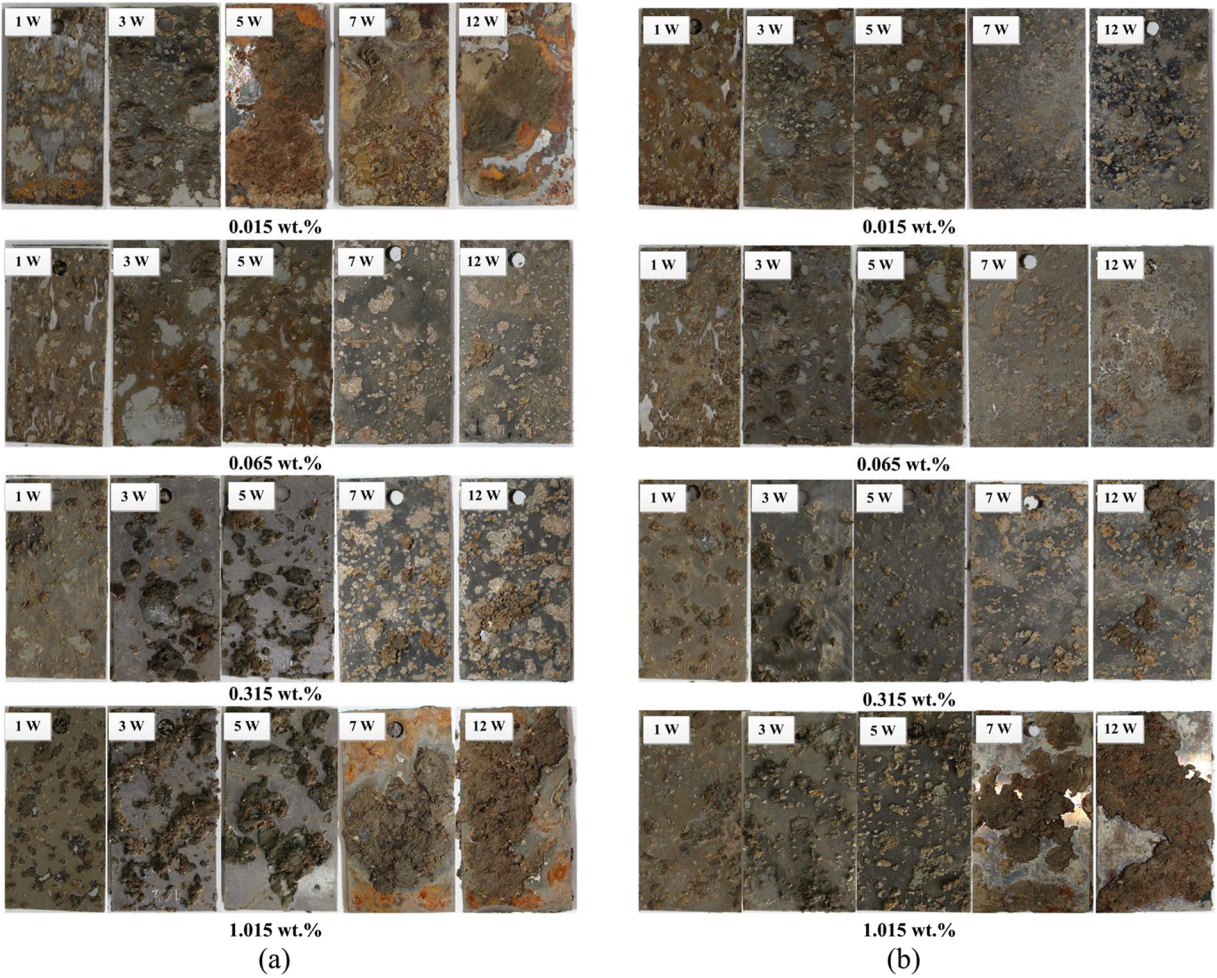
Ductile iron (**a**) and carbon steel (**b**) coupons exposed to soils of different chloride concentrations, i.e. 0.015%, 0.065%, 0.315%, 1.015% (wt.%) after 1, 3, 5, 7, 12 weeks.

The surface of ductile iron exposed to soils with different chloride contents gradually transferred from an initial grey-brown appearance to red-brown or yellow-brown after longer exposure (Fig. 2a; Supplementary Fig. S2a). During the exposure to soils of 0.015 wt.% chloride, the propagation of corrosion was observed with the increasing thickness of rust layers and expanding corrosion area dominated by general corrosion. By increasing the chloride to 0.065 wt.%, although general corrosion still occupied the major area for the first 5 weeks, pitting corrosion was obviously detected after 7 weeks of exposure. Similar transformations from general to pitting corrosion continued as the chloride increased. At high chloride contents such as 1.015 wt.%, the ductile iron even suffered from pitting corrosion as soon as the exposure initiated.

Under paralleled exposure conditions, the carbon steel performed relatively different from ductile iron in both of the surface color and corrosion shape (Fig. 2b; Supplementary Fig. S2b). The surface appearance gradually changed from light brown to dark green-grey, followed by uneven dark brown after longer exposure. Unlike the general corrosion of ductile iron, small but substantial pitting corrosion was observed at the initiation even when coupons were exposed to soils of low chloride concentrations. This phenomenon might indicate that carbon steel was more susceptible to chloride attack which was the major cause of localized corrosion of ferrous metals^7^. Interestingly, the localized corrosion area was more easily covered by cohesive soil particles, which might in turn aggravate the pitting corrosion.

Collectively speaking, the results suggested that general corrosion usually appeared in low chloride soil environments, while higher chloride concentrations tend to cause pitting corrosion. However, the propagation of localized corrosion, by expanding the discrete spots to continuous area, led to a corroded surface similar to general corrosion indicating that no strict boundaries of corrosion form existed under the complicated soil environment. Comparing the corrosion behavior of ductile iron and carbon steel, it showed that they presented different corrosion development in terms of general or localized corrosion. Ductile iron developed general corrosion at low chloride concentrations, which was absent from carbon steel, at early stage of exposure. Similar results in literature showed that chloride exerted more remarkable influence on corrosion of carbon steel than ductile iron^33^. Melchers^34^ also reported that the long-term corrosion rate of cast iron is only 10% of that for steel materials when exposed to marine and atmospheric environments. However, for both ferrous metals, corrosion continuously propagated to more serious levels with higher chloride concentrations and prolonged exposure time.

### Microstructure analysis

**Figure 3:**
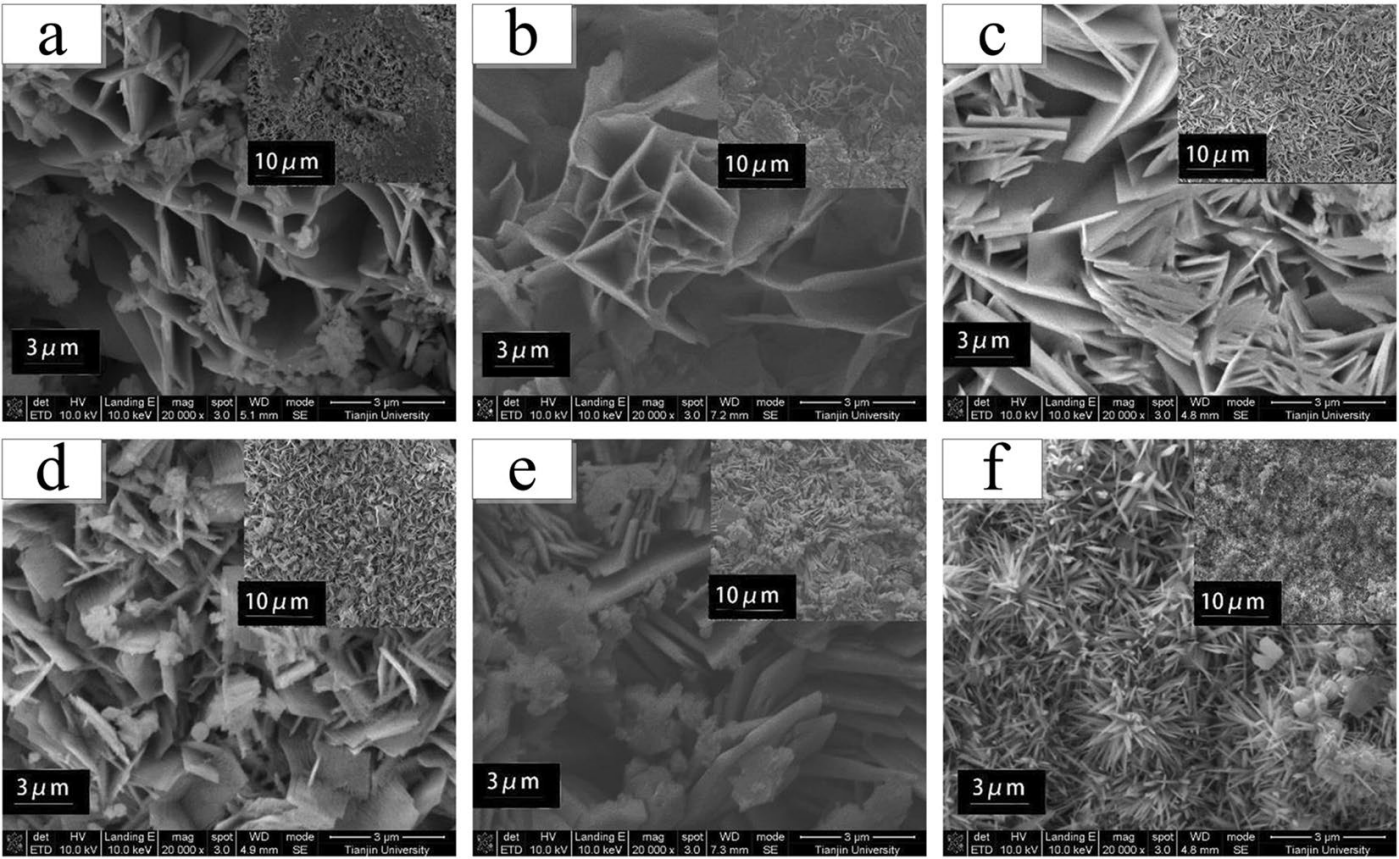
Rust surface of ductile iron coupons exposed to soils of different chloride concentrations i.e. (**a**) 0.015%, (**b**) 0.065%, (**c**) 0.115%, (**d**) 0.315%, (e) 0.515%, (**f**) 1.015% (wt.%) after 12 weeks.

**Figure 4:**
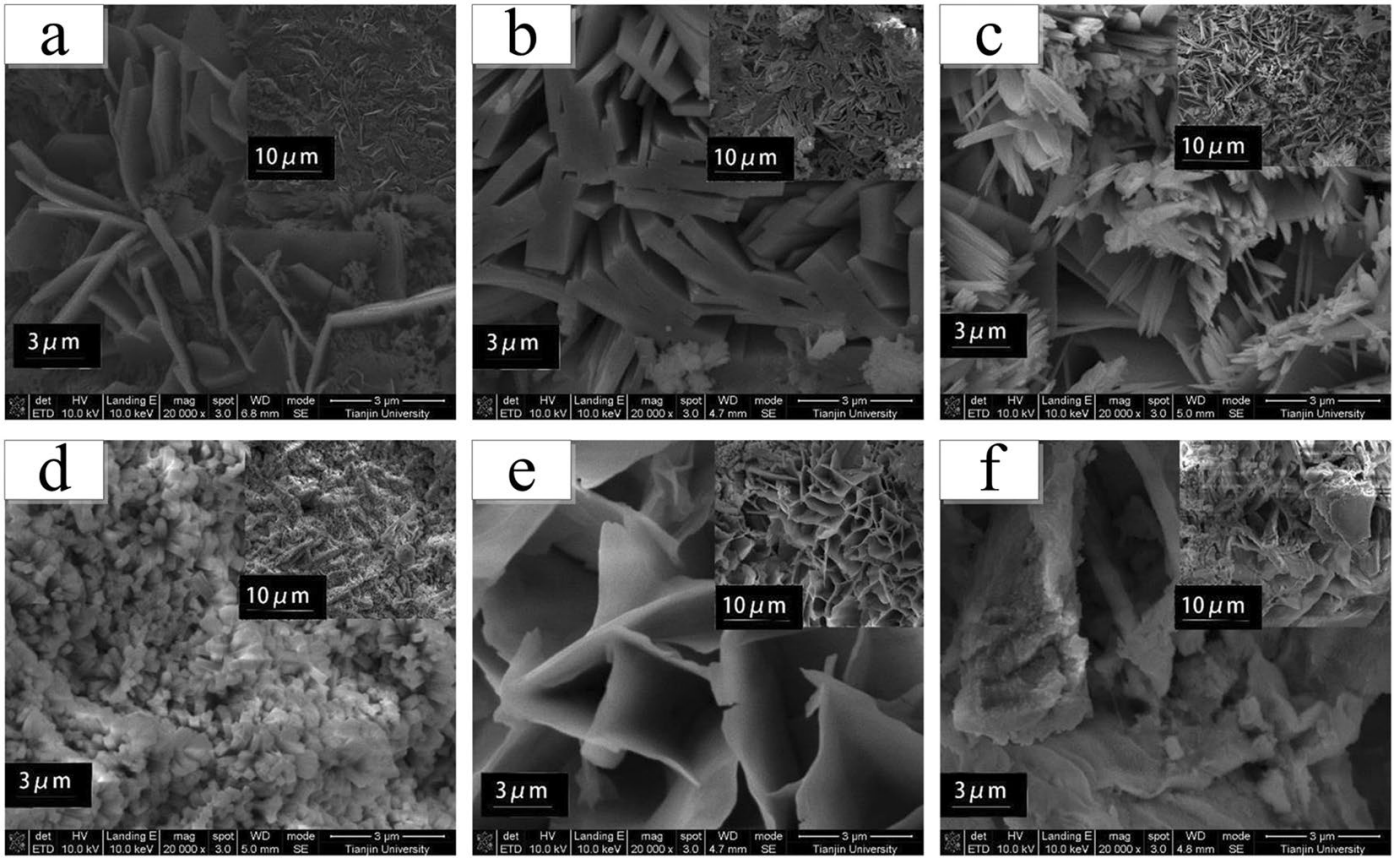
Rust surface of carbon steel coupons exposed to soils of different chloride concentrations i.e. (**a**) 0.015%, (**b**) 0.065%, (**c**) 0.115%, (**d**) 0.315%, (**e**) 0.515%, (**f**) 1.015% (wt.%) after 12 weeks.

To reveal the effects of chloride ions on corrosion products of ferrous metals, SEM measurements were conducted after 12 weeks of exposure for ductile iron and carbon steel as shown in Figs 3 and 4, respectively. For ductile iron exposed to relatively low chloride soils, porous and honeycomb-like structures were observed in Fig. 3a,b. Different from the typical appearance of oxyhydroxides which are one the most common corrosion products^35^, the rust layer might consist of other products such as ferric oxides. Besides, some disperse and fine globular crystals, probably identified as goethite (α-FeOOH)^15, 36, 37^ were observed in the cavities of the honeycomb-like structures. As the chloride concentrations increased, the scale-like and micaceous corrosion products gradually appeared (Fig. 3c). As Smith and McEnaney^38^ reported before, the plat-like morphology was possibly correlated to the formation of lepidocrocite (γ-FeOOH). A similar phenomenon was also documented by Ma *et al*.^15^ by investigating the surfaces of carbon steel exposed to marine and industrial environments. However, the crystalline examples of lepidocrocite (γ-FeOOH) could also form in other ways such as flowery and sandy structures^36^, indicating that the characterizations of corrosion products were always diverse and non-defined, largely depending on the surrounding exposure conditions. With the continuous enhancement of chloride contents, the micaceous particles partially grew to fine cotton ball structures in the cavity regions, possibly implying the transformation from lepidocrocite (γ-FeOOH) to goethite (α-FeOOH) (Fig. 3d). This phenomenon was in line with the previous report that out of all oxyhydroxides, α-FeOOH is the most stable formation; lepidocrocite (γ-FeOOH) could be further oxidized to goethite (α-FeOOH) provided that suitable exposure conditions are offered^39^. Accordingly, more compact crystals composed of α-FeOOH nucleated on the aggregating γ-FeOOH structures, and even became interconnected in Fig. 3e. However, different micro-morphology from the aforementioned images was also observed in Fig. 3f, somehow verifying that crystal structures of iron corrosion products often exhibited various characteristics even in unknown shapes.

For carbon steel coupons at low chloride levels, the micro-morphology of the corrosion products presented different characteristics (Fig. 4). When chloride concentrations were 0.015 wt.% and 0.065 wt.% (Fig. 4a,b), the micaceous and plat-like crystalline structures were already observed, as an indicator of the formation of γ-FeOOH in rust layers. Also, it showed that the scale-like layer was very porous interweaved with some small crystalline globules composed of α-FeOOH. The coexistence of γ-FeOOH and α-FeOOH in rust layers, presented in both of Figs 3d,e and 4a,b, altogether revealed the interaction between different oxyhydroxides and their transformation to one another as chloride contents increased. Further, as shown in Fig. 4c,d, the scale-like rust evidently became interconnected and compact with clubbed or spherical particles probably made of α-FeOOH. Therefore, it suggested that higher chloride concentrations remarkably facilitated the transformation from γ-FeOOH to α-FeOOH. Additionally, the discrete distribution of the spherical particles also implied that the structure evolution was incomplete and not uniform as well. As the chloride contents continued to rise, no obvious micaceous structures were observed anymore (Fig. 4e,f). Instead, the porous and honeycomb-like structures re-emerged and a sandy structure with dispersed cavities appeared uniquely.

From the aforementioned analysis, it is likely that γ-FeOOH and α-FeOOH were dominant in most of the rust species, usually in the form of porous micaceous and fine globular crystals structures, respectively. The coexistence of these two oxyhydroxides and their transformation to one another were somehow influenced by the variations in chloride concentrations.

### Composition analysis

To evaluate the crystalline characteristics of corrosion products, XRD measurements were carried out after 12 weeks of exposure (Tables 3–4, Supplementary Fig. S3-4). The results showed that quartz (SiO_2_) existed in all rust layers, probably attributable to the cohesive soil particles that tightly adhered to the corrosion sites (Fig. 2). The major existence of α-FeOOH and γ-FeOOH was also confirmed, which verified the SEM analysis and the surface color of rusts, knowing that α-FeOOH is yellow to brownish and γ-FeOOH is orange^35^. Further, the variations in rust compositions were observed as the chloride increased gradually. For ductile iron at 0.015 wt.% of chloride, the rusts were mainly composed of α-FeOOH and iron oxides (mainly magnetite (Fe_3_O_4_)). At higher chloride situations, γ-FeOOH and β-Fe_8_O_8_(OH)_8_Cl_1.35_ gradually appeared along with the re-emergence of α-FeOOH. Interestingly, different but still analogous patterns were found in the rust layers of carbon steel coupons. At the low chloride content of 0.015 wt.%, the rust was primarily composed magnetite (Fe_3_O_4_); as chloride increased, γ-FeOOH and β-Fe_8_O_8_(OH)_8_Cl_1.35_ started to appear, but the former proceeded to disappear since the chloride reached as high as 0.515 wt.%. Presumably, the corrosion products of both ductile iron and carbon steel were influenced by the level of chloride concentrations, which in turn reflected the variations in corrosion reactions under different chloride situations. Accordingly, potential transformations of corrosion products under low and high chloride concentrations were classified as follows.

**Table 3:** Main compositions of corrosion products of ductile iron coupons exposed to soils of different chloride concentrations after 12 weeks.

Corrosion products		Chloride concentrations (wt.%)					
		0.015	0.065	0.115	0.315	0.515	1.015
Quartz	SiO_2_	√	√	√	√	√	√
Wustite	FeO	√	√				
Lepidocrocite	γ-FeOOH		√	√	√	√	
β-Fe_8_O_8_(OH)_8_Cl_1.35_			√	√	√		
Goethite	α-FeOOH	√				√	√
Magnetite	Fe_3_O_4_	√	√	√	√	√	
Hematite	α-Fe_2_O_3_		√	√	√

**Table 4:** Main compositions of corrosion products of carbon steel coupons exposed to soils of different chloride concentrations after 12 weeks.

Corrosion products		Chloride concentrations (wt.%)					
		0.015	0.065	0.115	0.315	0.515	1.015
Quartz	SiO_2_	√	√	√	√	√	√
Lepidocrocite	γ-FeOOH		√	√	√		
β-Fe_8_O_8_(OH)_8_Cl_1.35_		√	√	√	√	√	
Goethite	α-FeOOH					√	√
Magnetite	Fe_3_O_4_	√	√	√	√	√	√
Hematite	α-Fe_2_O_3_						√

When exposed to low chloride soils, iron oxides and goethite (α-FeOOH) were mainly observed. Of all the oxyhydroxide types, goethite (α-FeOOH) is identified as the most stable phase^39^, which is more likely produced by subsequent oxidization of other unstable products such as lepidocrocite (γ-FeOOH) and akaganite (β-FeOOH)^15, 21^. In this case, however, no other oxyhydroxides except for goethite was detected in the rust layers of ductile iron at chloride of 0.015 wt.%. It implies that the stable phase of goethite (α-FeOOH) developed directly when ferrous metals were exposed to low chloride soils. This hypothesis correlated well with the previous study which was in a situation completely free of salinity (such as rural environments)^40^. In addition, further dehydration and crystallization of goethite (α-FeOOH) might change to non-hydrated iron oxides, such as hematite (α-Fe_2_O_3_) shown in Tables 3–4. For the formation of magnetite (Fe_3_O_4_), the insufficient oxygen supply was probably the reason. Similarly, previous studies also found substantial magnetite in iron exposed for long periods in soils, the existence of which, in contrast, is not yet clear in atmospheric corrosion process^7^.

With the increasing chloride concentrations, lepidocrocite (γ-FeOOH) gradually evolved but only maintained at the intermediate regions between low and high levels of chloride. This phenomenon was followed by the lagging appearance of β-Fe_8_O_8_(OH)_8_Cl_1.35_, which was similar in molecular formula to the intermediate products mentioned by Ma *et al*.^15^. Still, it could be inferred that β-Fe_8_O_8_(OH)_8_Cl_1.35_ was an intermediate corrosion product that was primarily derived from γ-FeOOH and further developed into α-FeOOH. However, this unique β-formed crystal has never been specified in literature. Instead, the formation of akaganite (β-FeOOH), an unstable phase of iron oxyhydroxide, has been largely emphasized in traditional research under conditions of high chloride contents^9–12^. In contrary to previous studies, no obvious β-FeOOH was observed in this study for both ductile iron and carbon steel, even though high concentrations of chloride were provided. Two possible speculations are given here for the absence of β-FeOOH. First, it was possibly due to the inhomogeneous characteristics of soils such as the high diffusion resistance of chloride through soils, relatively different from the atmospheric and aquatic situations under which most of the conventional conclusions were drawn. According to Ma *et al*.^15^, β-FeOOH only appears when chloride ions are above the critical concentrations in atmospheric environments. In saline soils, perhaps, the already high chloride concentrations in soils are still difficult to continuously diffuse through the soil matrix and condense on the metal surface, thus the surface concentrations are still lower than the required critical concentrations. Secondly, the absence of β-FeOOH in this study might be attributed to the relatively short exposure time and the incomplete analysis of the rust compositions during the whole exposure period which requires further research.

**Figure 5:**
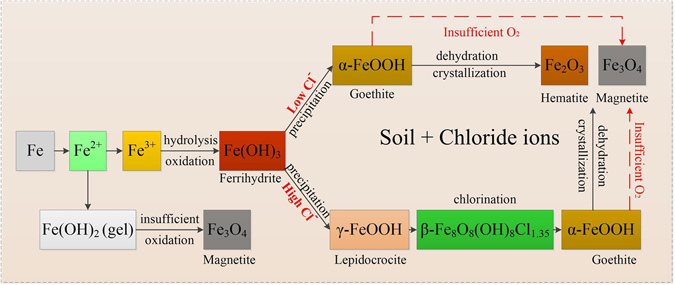
The potential transformation processes of ferrous metals in soils with different chloride contents.

Overall, it was reasonable to assume that chloride ions in soils significantly influenced the compositions of corrosion products, possibly by diverting the corrosion processes and even participating in the corrosion reactions, especially at high chloride concentrations. The potential transformation processes are shown in Fig. 5.

### Corrosion kinetics

The general corrosion rate based on the weight loss measurement is calculated by equation (1), where *v* is the general corrosion rate (mm/y), *W* is the weight loss (g), *ρ* is the density of ferrous metals (ductile iron 7.3 g/cm^3^, carbon steel 7.8 g/cm^3^), *A* is the total exposed area (cm^2^) and *T* is the exposure time (d).

1 $$v=\frac{W}{\rho AT}\times 365\times 10$$

**Figure 6:**
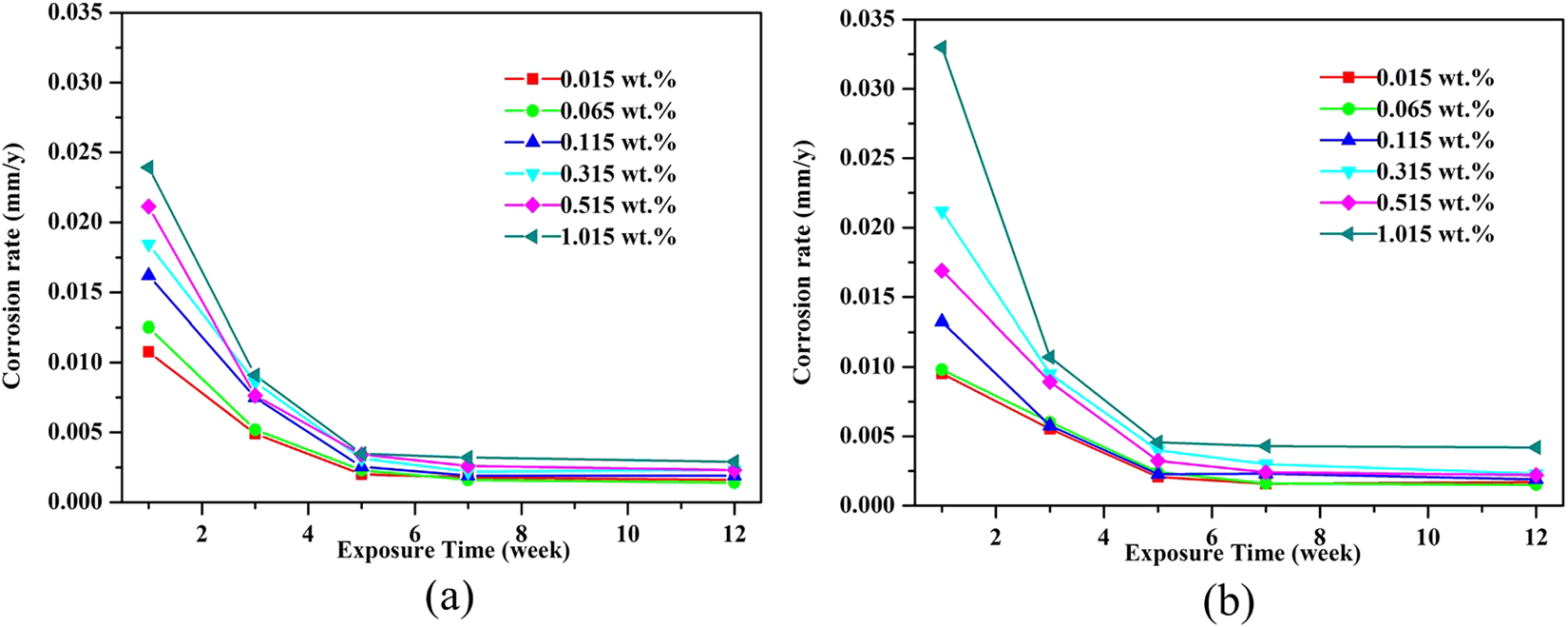
Corrosion rates of ductile iron (**a**) and carbon steel (**b**) after being exposed in soils with different levels of chloride.

The corrosion rates of ductile iron and carbon steel showed similar trends after different exposure periods (Fig. 6). Regardless of the variation in chloride levels, the corrosion rates decreased sharply from the initiation till five weeks of exposure; since then the reduction in corrosion rates was less pronounced until a relatively stable status was achieved. Meanwhile, with the enhancement of chloride concentrations, the corrosion rates generally increased but the accelerating effect weakened as the exposure time prolonged. Further, the acceleration influence of chloride on corrosion rates was more evident in carbon steel compared with ductile iron, indicating that carbon steel might be more susceptible to chloride attacks. This was also demonstrated by the local penetration pit depths in Table 5, showing that both of the maximum and average pit depths of carbon steel were larger than those of ductile iron. Similarly, the relatively low corrosion resistance of carbon steel against the localized attack by chloride ions was also verified by Ma *et al*.^15^. Besides, as chloride concentrations increased, the pit depths increased as well. As Caleyo *et al*.^41^ reported, the profile of pit depths largely reflects the severity of the threat posed by soil corrosion.

**Table 5:** Maximum and average pit depths of ductile iron and carbon steel coupons exposed to soils of different chloride concentrations after 12 weeks.

Material Types		Chloride concentrations (wt.%)					
		0.015	0.065	0.115	0.315	0.515	1.015
Ductile iron	Maximum pit depth /mm	0.57	0.72	0.69	0.97	1.02	1.28
	Average pit depth/mm	0.25	0.45	0.49	0.61	0.67	0.77
Carbon steel	Maximum pit depth/mm	0.73	0.80	0.87	1.09	1.21	1.46
	Average pit depth/mm	0.39	0.56	0.63	0.66	0.70	0.83

**Figure 7:**
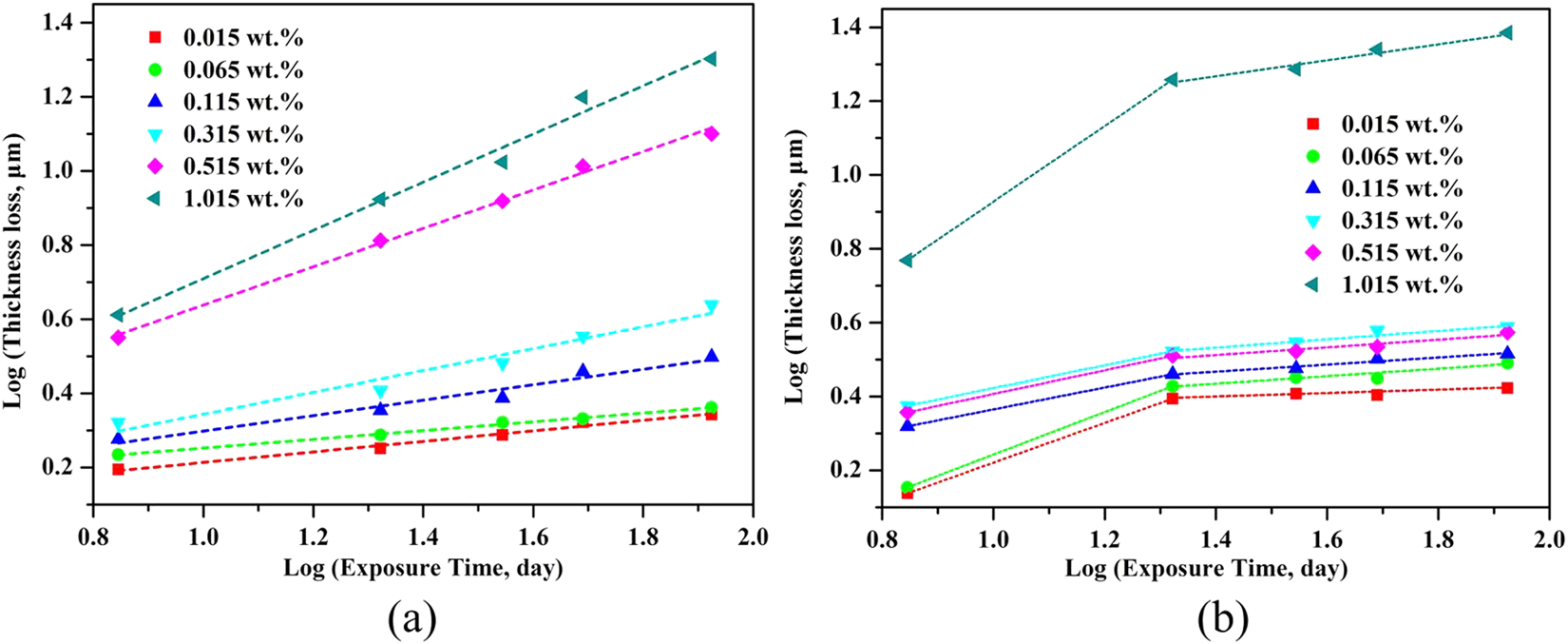
Bilogarithmic plots of thickness loss versus exposure time of ductile iron (**a**) and carbon steel (**b**) after being exposed in soils with different levels of chloride.

Figure 7 used the results of thickness loss derived from the weight loss measurement by plotting the thickness loss (μm) against the exposure time in log-log coordinates^15^. It was found that the corrosion process of ductile iron (Fig. 7a) fitted well with the probabilistic model proposed by Romanoff^42^ and widely utilized by other studies^7, 15, 43^, which was intended for estimating the failure of steel pipes based on equation (2):

2 $$D=k{t}^{n}$$

*D* is the loss of thickness, and *k* and *n* are regression parameters. This well-known formulation is widely adopted in decision making systems for rehabilitation and maintenance^20^, such as the UtilNets program developed by the European Union^37^. Nevertheless, the corrosion behavior of carbon steel (Fig. 7b) deviated from this classic equation (2); instead, the bi-logarithmic curve in this case was a broken line composed of two linear segments, which could be delineated based on the equation (3)^44^:

3 $$D=k{{t}_{1}}^{{n}_{1}-{n}_{2}}{t}^{{n}_{2}}(t\ge {t}_{1})$$

*t*_1_ is the length of the first period of slope *n*_1_, and *n*_2_ is the slope of the second period. The similar phenomenon was once reported by Ma *et al*.^15^ when carbon steel coupons were exposed to marine atmosphere. However, different from the changing turning points in previous research, the turning points in this study maintained at the week three regardless of the variations in chloride levels. However, the actual *t*_1_ might be shorter than three weeks due to lack of data points between week 1 and 3. The regression results are presented in Supplementary Table S2, according to the equation (2) and (3), respectively.

According to Li^45^ and Ma^15^, the corrosion rates increase when *n* > 1 and decrease while *n* < 1; n = 1 indicates that the corrosion proceeds at a constant rate. In this case, all slope values of *n* were much less than 1 except *n*_1_ of carbon steel exposed to soils of 1.015 wt.% chloride contents. This phenomenon was in line with the results in Fig. 6, indicating that corrosion of both ductile iron and carbon steel were decelerated processes as the exposure prolonged. Also, most of *n* values were even less than 0.5 implying that the corrosion was highly restrained. Likely, the corrosion in soil is more complicated than in atmospheric environments due to the soil conditions in particular the oxygen diffusion, the moisture content and the chloride ions reaching the surfaces of the buried metals. It has been reviewed that the soil moisture will be profoundly influenced by soil types ranging from 0.5% in sand soil to 217% for bentonite clay soil^7^. Accordingly, the oxygen diffusion will change as well, i.e. being lowest at highest moisture contents. In this case, 20% moisture as a normal status, the diffusion of oxygen might be the limiting factor, because the soil samples were filled in a relatively compact way and no aeration process was provided in the well-sealed soil reactors. On the other hand, as the exposure prolonged, the thickening and compacting rust layers primarily composed of γ-FeOOH, β-Fe_8_O_8_(OH)_8_Cl_1.35_, α-FeOOH and α-Fe_2_O_3_ may also contribute to inhibiting the corrosion process. However, to verify the correlation between different corrosion products and corrosion rates, more quantitative analysis of corrosion products and their evolution with time is needed.

Moreover, for ductile iron, *n* generally increased with the enhancement of chloride contents, probably indicating that the decreasing of corrosion rates become less severe. Together with the results in Fig. 6a, it was reasonable to infer that higher chloride contents not only induced higher corrosion rates but also suppressed the decreasing of corrosion for ductile iron, especially during the corrosion initiation. Chloride ions, minor in radius, are assumed necessary for the corrosion initiation mainly due to its ability for de-passivation of the passive film^8^. Additionally, the increased chloride contents are conducive to diminishing soil resistivity thus providing a more favorable environment for corrosion. Nevertheless, for carbon steel, deviation from the classic function (2) was observed along with the first slope values *n*_1_ much bigger than the second slope values *n*_2_. Interestingly, the values of *n*_1_ generally decreased under intermediated chloride levels (0.115–0.515 wt.%). According to the previous investigation^15^, this phenomenon could be possibly attributed to the aforementioned potential transformation from γ-FeOOH to α-FeOOH that made the rust layer more stabilized. However, as chloride reached 1.015 wt.%, *n*_1_ abruptly increased to almost 1 implying that the rust again presented less protective properties. The reason for this irregular fluctuation of corrosion behavior was still not clear which needs further study by quantitatively analyzing the corrosion products and their transformation.

Collectively speaking, corrosion of ferrous metals in soils is a decelerated process, but chloride ions can suppress the decreasing for both ductile iron and carbon steel following different kinetics models respectively. By comparing the two ferrous metals, we observed that chloride could increase more corrosion on carbon steel than the ductile iron. Further, after reaching a certain chloride degree, the thickness loss would increase remarkably for both of ductile iron and carbon steel (Fig. 7), with different critical chloride concentrations (ductile iron 0.515 wt.%, carbon steel 1.015 wt.%). Accordingly, this study suggest that ductile iron might be advantageous over carbon steel in terms of corrosion resistance, when it is used as pipe materials in saline soils with high levels of chloride. However, other features including the mechanical and economical properties of those materials require further verification.

### Linear polarization measurements

**Figure 8:**
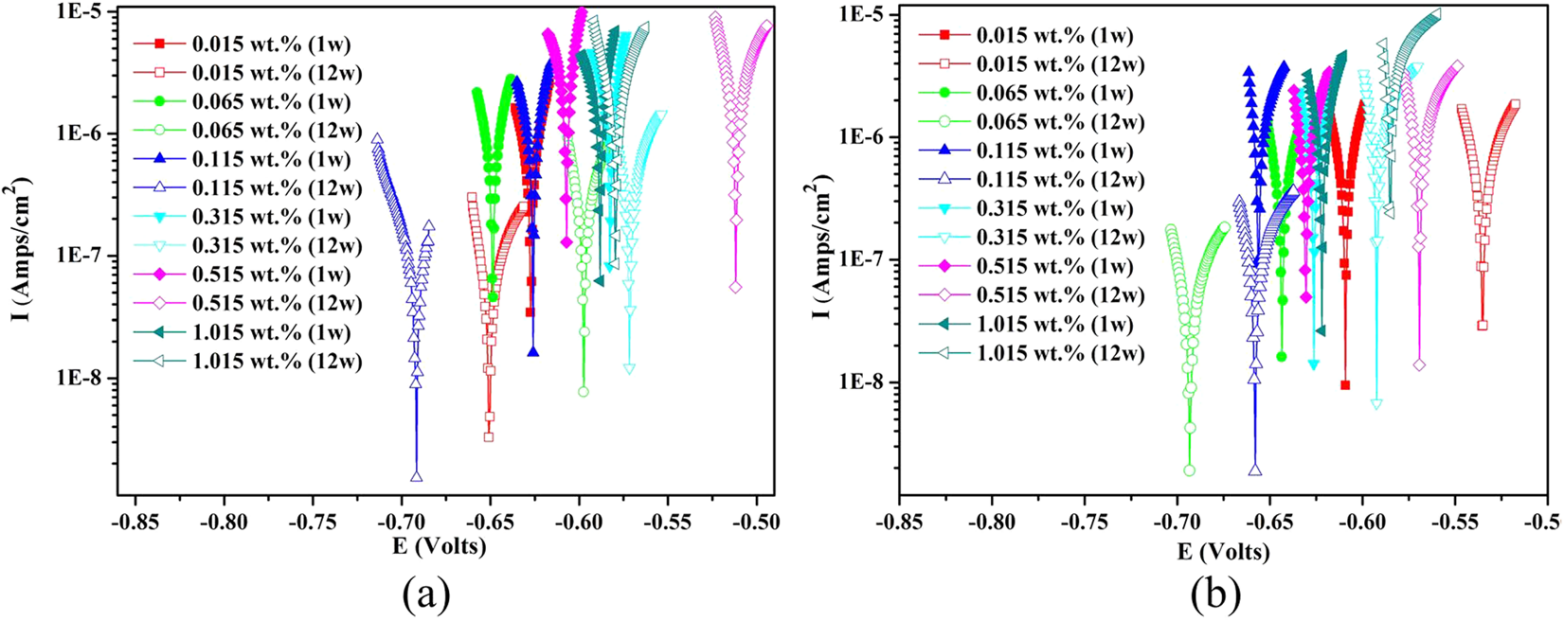
Polarization curves i.e. potential-log (current) of ductile iron (**a**) and carbon steel (**b**) in soils with different levels of chloride after 1 week (solid) and 12 weeks (hollow) of exposure.

**Figure 9:**
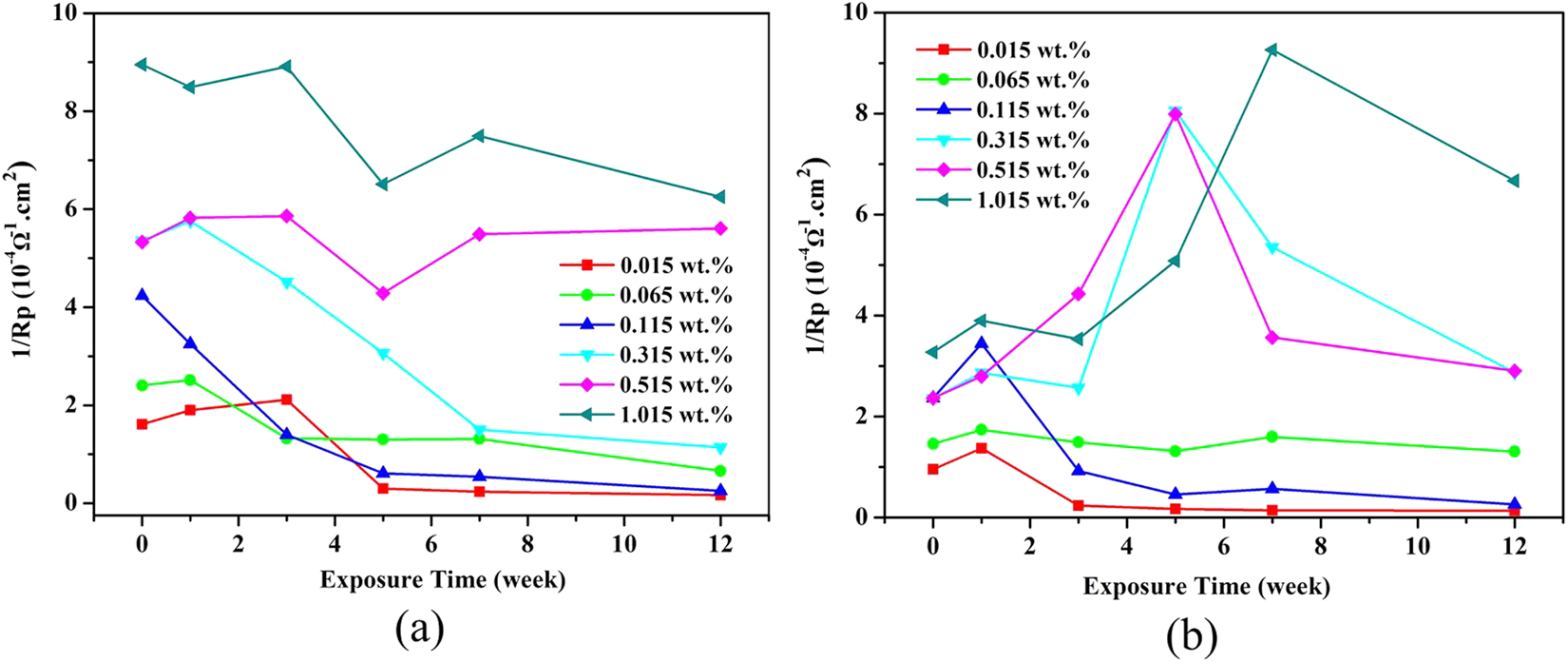
The variation of 1/*R*p of ductile iron (**a**) and carbon steel (**b**) in soils with different levels of chloride.

Figure 8 shows the typical linear polarization curves at the initiation (week 1) and end of exposure (week 12); other curves not shown here performed similarly and were used to calculate the reciprocal of linear polarization resistance (*R*p) (Fig. 9). Generally, most coupons presented higher potentials after 12 weeks exposure which indicated a more stabilized state, knowing that lower *E*_corr_ values suggest a higher corrosion risk^46, 47^. However, the fluctuating trend of potentials as the chloride increased implied that the corrosion process in soils was complicated and the corrosion potentials were difficult to predict. Figure 9 presented the variations in the reciprocal of linear polarization resistance (*R*p), which is positively proportional to the instantaneous corrosion rate^15, 46, 47^. Different from the clear trend of corrosion rates (Fig. 6), the instantaneous corrosion rates based on polarization measurements showed higher variation, probably attributed to the unsteady electrochemical reactions that induced the transformation of varied corrosion products. In particular, for carbon steel, as the chloride contents reached beyond 0.315 wt.%, the values of 1/ *R*p fluctuated with the exposure time, which was likely caused by the variation in rust layers. The changing status of lepidocrocite (γ-FeOOH) and β-Fe_8_O_8_(OH)_8_Cl_1.35_ that are able to exhibit reduction reactivity may enhance transient corrosion processes, thus leading to the increase of 1/*R*p to some degree. Generally, the trends of instantaneous corrosion rates in terms of *1/R*p, decreasing with time and increasing with chloride contents, roughly correspond with the general corrosion trends in Fig. 6., and it also compensated the incapability of general corrosion rates to detect the *in-situ* corrosion transformation.

### Electrochemical impedance spectroscopy

**Figure 10:**
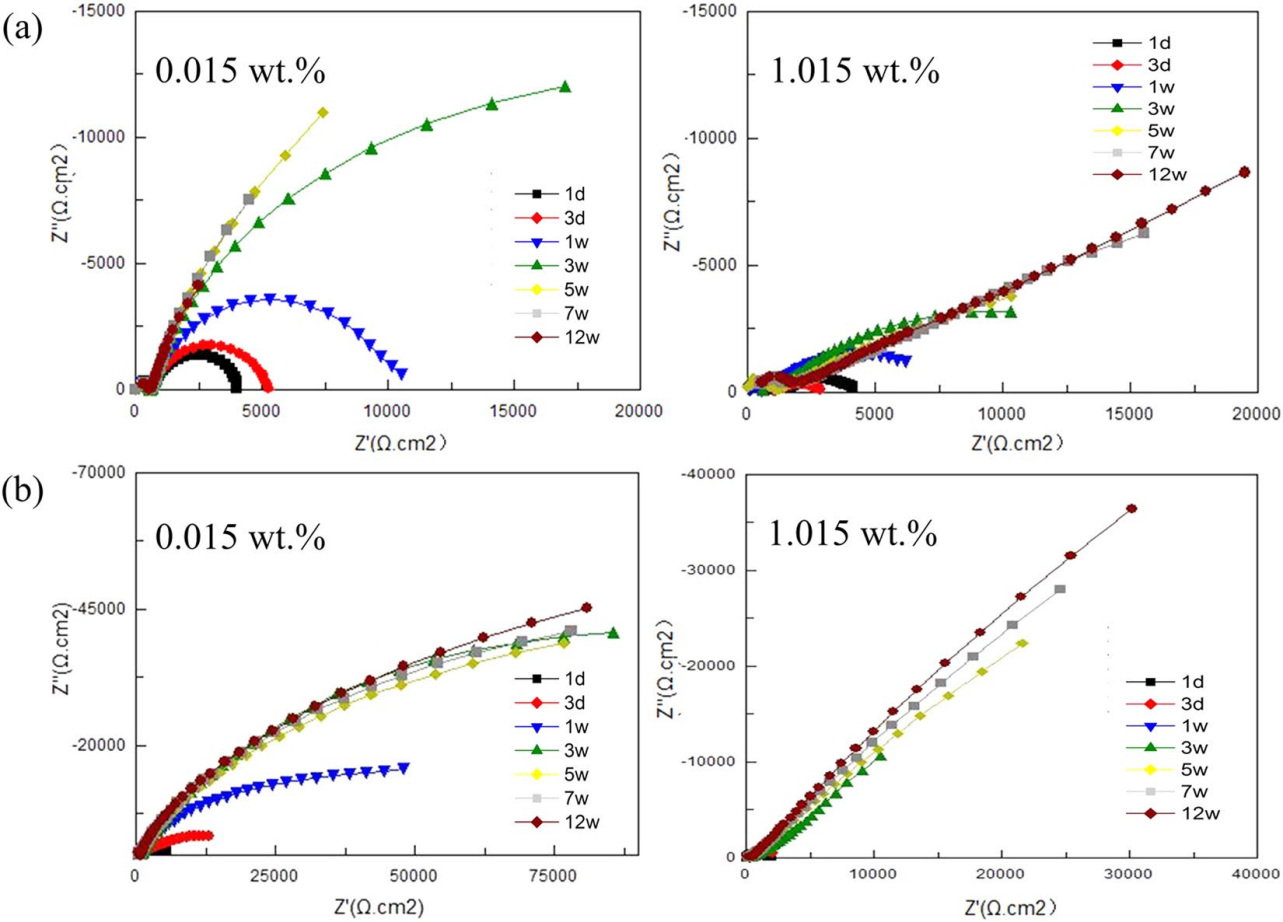
Nyquist plots of ductile iron (**a**) (the first row) and carbon steel (**b**) (the second row) in soils (0.015 wt.% and 1.015 wt.% of chloride contents) after different exposure periods.

EIS measurements of ductile iron and carbon steel were carried out after exposure to different chloride enriched soils. Figure 10 shows the representative Nyquist plots corresponding to the lowest (0.015 wt.%) and highest (1.015 wt.%) chloride conditions. In general, two semi-circles related to two constant-phase elements (CPE) were observed in each plot, representing the electrical double layers (EDL) between soils and rusts, and between rusts and metal surfaces, respectively. The second semi-circle, in relatively low frequency range, was the main focus which exhibited the behavior of rust layers.

When exposed to low chloride soils (0.015 wt.%), the second semi-circle expanded largely with the exposure time, which implied the increasing resistivity of the rust layers probably caused by the growth in rust thickness and density. Likely, this phenomenon explained why the corrosion rates of coupons decreased with exposure time, assuming that the rust layers behaved as a protective layer against corrosion acceleration. On the other hand, the impedance diagrams obtained in the spectra were not perfect semicircles, indicating the heterogeneities of the rust layers^31, 48^. This non-monotonous characterization was in line with the morphology analysis in sections 3.1 and 3.2, that the development of the rust layers was not even or monotonous. When exposed to soils of high chloride contents (1.015 wt.%), the electrochemical behavior of rust layers was relatively different. As the exposure time increased, the second semi-circle gradually transformed into diffusional tails, indicating the increasing difficulty in diffusion process. Likely, this performance was attributable to higher chloride concentrations which facilitated the continuous thickening and compacting of the rust layer, thus making the corrosion process limited by the diffusion process. Similar studies reported that the building up of rust layers may decrease the corrosion rates, apparently by blocking the pathways for oxygen diffusion and limiting cathodic reactions^7^. Therefore, it could be inferred that chloride ions exert multiple effects on the corrosion kinetics of ferrous metals, not only by suppressing the decreasing of corrosion rates at the initiation, but also diverting corrosion reactions and influencing the thickness and density of corrosion layers. In addition, in-depth analysis of the electrochemical results including the polarization curves, EIS and equivalent electrical circuits need to be further conducted for the understanding of the corrosion development and processes.

## Conclusions

The effects of chloride ions in saline soils on corrosion of ductile iron and carbon steel were investigated in terms of surface morphology, rust compositions and corrosion kinetics. This has led to the following key findings:

1. Low levels of chloride tend to cause general corrosion while high levels of chloride likely induce localized corrosion. However, no strict boundaries exist between the general and localized process along with higher chloride concentrations and prolonged exposure time.
2. Chloride influences the compositions of corrosion products by diverting potential corrosion pathways or even participating in corrosion reactions directly. At low levels of chloride, α-FeOOH and iron oxides are major corrosion products; at high levels of chloride, γ-FeOOH and β-Fe_8_O_8_(OH)_8_Cl_1.35_ appears sequentially. β-Fe_8_O_8_(OH)_8_Cl_1.35_ is observed for the first time when high chloride concentrations are provided, and Fe_3_O_4_ exists in most of the rust layers.
3. Corrosion of ductile iron and carbon steel in soils is decelerated, but higher chloride concentrations induce higher corrosion rates, larger pit depths and suppress the decreasing of corrosion rates especially during the initiation. After longer exposure, however, high levels of chloride may thicken the rust layers, thus retarding corrosion conversely. The instantaneous corrosion rates based on *in-situ* polarization measurements largely conforms to the weight-loss based corrosion rates.
4. Compared with ductile iron, chloride could increase more corrosion on carbon steel which is also more susceptible to localized attacks than ductile iron. The corrosion kinetics of ductile iron corresponds well with the classic probabilistic model, whereas that of carbon steel follows the bilinear model.

## Electronic supplementary material

Supplementary Information
