# Supplementary material for: Effects of chloride ions on corrosion of ductile iron and carbon steel in soil environments
Source: Sci Rep. 2017 Jul 31;7:6865. doi: 10.1038/s41598-017-07245-1 (PMC5537275; doi:10.1038/s41598-017-07245-1)
Supplement: Supplementary file 1 — Supplementary Information [file 41598_2017_7245_MOESM1_ESM.docx]

**Supplementary Information**

**Journal**: Scientific Reports

**Title**: Effects of chloride ions on corrosion of ductile iron and carbon steel in soil environments

**Authors**: Yarong Song^a,b^, Guangming Jiang^b^, Ying Chen^a^, Peng Zhao^a*^, Yimei Tian^a,c*^

^a^ *School of Environmental Science and Engineering, Tianjin University, Tianjin 300350, China*

^b^*Advanced Water Management Centre, The University of Queensland, St. Lucia, Brisbane QLD 4072, Australia*

^c^ *Tianjin Engineering Center of Urban River Eco-Purification Technology, Tianjin 300350, China*

*Corresponding author. Tel: +86 13920975826.

E-mail address: [zhpeng@tju.edu.cn](mailto:zhpeng@tju.edu.cn) (P. Zhao).

*Corresponding author. Tel: +86 15122638980.

E-mail address: [ymtian_2017@163.com](mailto:ymtian_2017@163.com) (Y.M. Tian).

| Parameters | Value | Parameters | Value |
| --- | --- | --- | --- |
| pH | 6.98 | Electrical conductivity (μs/cm) | 2.8 |
| Turbidity (NTU) | 0.00 | Total dissolve solids (mg/L) | 35 |
| NH_3_-N | 0.0006% | Chloride | 0.0002% |

**Table S1. Main parameters of distilled water quality.**

**
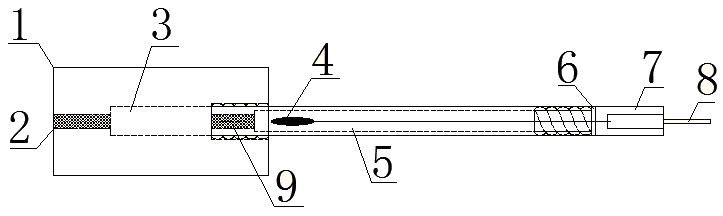
**

**Figure S1.** The reference electrode (1&7: polytetrafluoroethylene cover; 2&9: porous sand core; 3: saturated electrolytic cavity; 4: Ag-AgCl; 5: electrolyte cavity; 6: seal ring; 8: copper conductor).

**
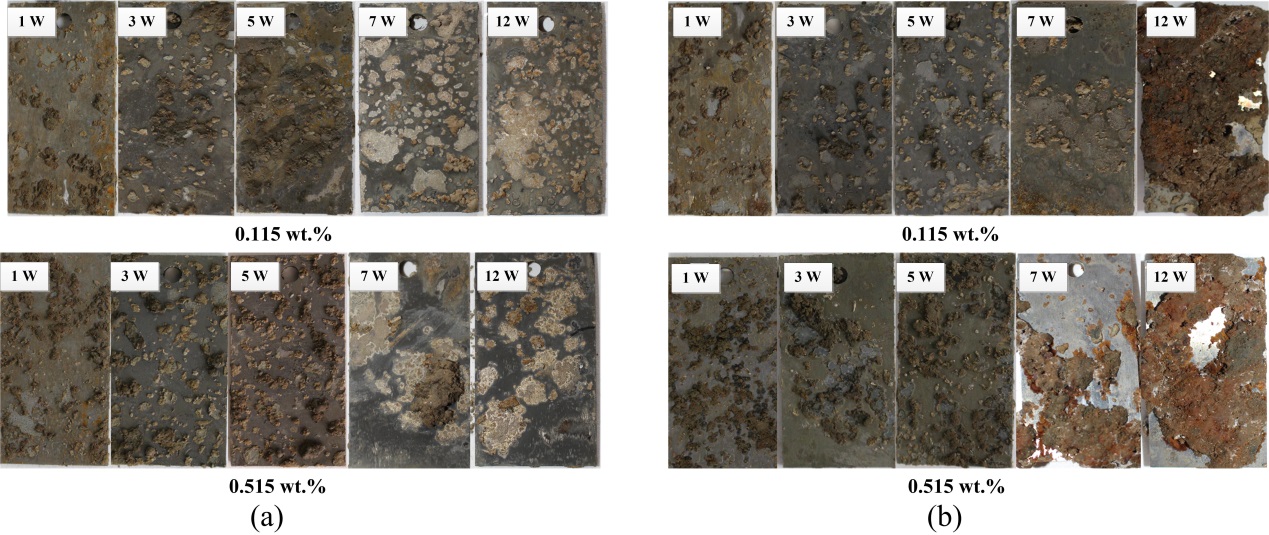
**

**Figure S2.** Ductile iron (a) and carbon steel (b) coupons exposed to soils of different chloride concentrations, i.e. 0.115%, 0/515% (wt.%) after 1, 3, 5, 7, 12 weeks.


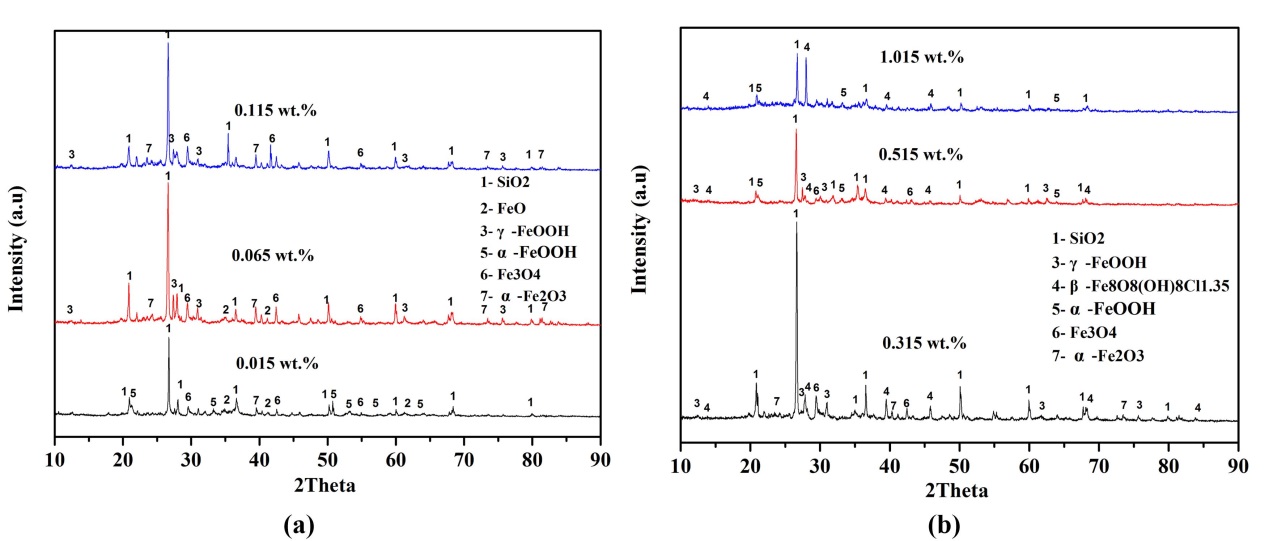


**Figure S3.** XRD analysis of ductile iron coupons exposed to soils of different chloride concentrations, i.e. (a) 0.015%, 0.065%, 0.115%, (b) 0.315%, 0.515%, 1.015% (wt.%) after 12 weeks.


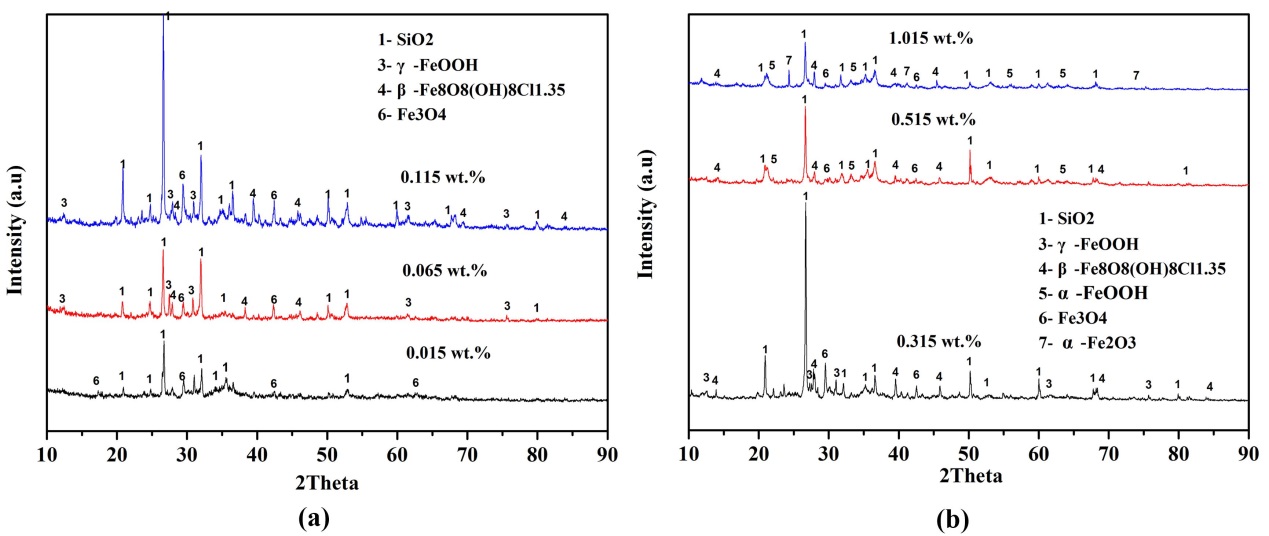


**Figure S4.** XRD analysis of carbon steel coupons exposed to soils of different chloride concentrations, i.e. (a) 0.015%, 0.065%, 0.115%, (b) 0.315%, 0.515%, 1.015% (wt.%) after 12 weeks.

| **Chloride contents** | **Ductile iron** | | | **Carbon steel** | | | |
| --- | --- | --- | --- | --- | --- | --- | --- |
| wt.% | k | n | R2 | k | n1 | n2 | R2 |
| 0.015 | 1.178 | 0.142 | 0.982 | 0.482 | 0.538 | 0.0467 | 0.968 |
| 0.065 | 1.362 | 0.118 | 0.995 | 0.465 | 0.575 | 0.101 | 0.939 |
| 0.115 | 1.227 | 0.209 | 0.950 | 1.172 | 0.296 | 0.0959 | 0.986 |
| 0.315 | 1.118 | 0.295 | 0.944 | 1.298 | 0.310 | 0.113 | 0.880 |
| 0.515 | 1.322 | 0.517 | 0.995 | 1.216 | 0.322 | 0.105 | 0.966 |
| 1.015 | 1.147 | 0.650 | 0.985 | 0.799 | 1.025 | 0.216 | 0.942 |
| Function | $D=kt^{n}$ | | | $D=k{t_{1}}^{n_{1}-n_{2}}t^{n_{2}}$ | | | |

**Table S2.** **Regression coefficients of the corrosion data for ductile iron and carbon steel.**
